# Effects of Triterpene Soyasapogenol B from *Arachis hypogaea* (Peanut) on Differentiation, Mineralization, Autophagy, and Necroptosis in Pre-Osteoblasts

**DOI:** 10.3390/ijms23158297

**Published:** 2022-07-27

**Authors:** Hyung-Mun Yun, Joon Yeop Lee, Soo Hyun Kim, Il Keun Kwon, Kyung-Ran Park

**Affiliations:** 1 Department of Oral and Maxillofacial Pathology, School of Dentistry, Kyung Hee University, Seoul 02447, Korea; yunhm@khu.ac.kr; 2 National Development Institute for Korean Medicine, Gyeongsan 38540, Korea; chool9090@nikom.or.kr (J.Y.L.); beluga81@nikom.or.kr (S.H.K.); 3 Department of Dental Materials, School of Dentistry, Kyung Hee University, Seoul 02447, Korea; kwoni@khu.ac.kr; 4 Gwangju Center, Korea Basic Science Institute (KBSI), Gwangju 61751, Korea

**Keywords:** *Arachis hypogaea*, peanut, osteoblast, RUNX2, Soyasapogenol B

## Abstract

Triterpenes are a diverse group of natural compounds found in plants. Soyasapogenol B (SoyB) from *Arachis hypogaea* (peanut) has various pharmacological properties. This study aimed to elucidate the pharmacological properties and mechanisms of SoyB in bone-forming cells. In the present study, 1–20 μM of SoyB showed no cell proliferation effects, whereas 30–100 μM of SoyB increased cell proliferation in MC3T3-E1 cells. Next, osteoblast differentiation was analyzed, and it was found that SoyB enhanced ALP staining and activity and bone mineralization. SoyB also induced RUNX2 expression in the nucleus with the increased phosphorylation of Smad1/5/8 and JNK2 during osteoblast differentiation. In addition, SoyB-mediated osteoblast differentiation was not associated with autophagy and necroptosis. Furthermore, SoyB increased the rate of cell migration and adhesion with the upregulation of MMP13 levels during osteoblast differentiation. The findings of this study provide new evidence that SoyB possesses biological effects in bone-forming cells and suggest a potentially beneficial role for peanut-based foods.

## 1. Introduction

Bone development, formation, and regeneration are regulated by a complex process involving the differentiation of osteoblasts that are derived from mesenchymal stem cells (MSCs) [1]. Osteoblast differentiation and migration are required to form new bone and remodel old bone through the synthesis and secretion of the organic component of the bone matrix and bone matrix mineralization comprising hydroxyapatite [2,3]. The multiple signaling pathways associated with BMP2 and Wnt3a proteins tightly regulate osteoblast differentiation by upregulating the expression and activity of RUNX2, which is a core transcription factor in osteoblast differentiation [4,5]. Pathologically, the impairment and dysregulation of osteoblast differentiation lead to the pathogenesis of bone diseases such as osteoporosis and periodontitis [6,7]. Accordingly, the compounds targeting osteoblast differentiation are attracting attention as a promising strategy for the prevention and treatment of bone diseases.

Natural compounds obtained from plants have been widely used in traditional medicines to treat diseases, including osteoporosis [8]. A member of the legume family (*Fabaceae*), *Arachis hypogaea*, commonly known as the peanut, is widely cultivated (over about 25.5 million hectares) as a major oilseed and as food for human consumption worldwide [9]. *A. hypogaea* is also used as a traditional medicine with neuroprotective, anti-oxidative, and anti-obesity properties, and extracts of *A. hypogaea* contain bioactive compounds including triterpene, isoflavones, resveratrol, and polyphenols [9,10,11]. Soyasapogenol B (SoyB), a triterpene, has been reported on for its biological effects and mechanisms, including anti-viral, anti-cancer, anti-inflammatory, and hepatoprotective activities [12,13,14]. Recently, it was reported that germinated soy germ with increased soyasaponin Ab protects against osteoporosis [15]. However, the biological activity and mechanism of SoyB have not been reported in bone-forming cells yet.

In the present study, we isolated a pure triterpene saponin compound, SoyB (>99.9% purity), from *A. hypogaea* fruits, and initially examined its pharmacological properties in cell proliferation in pre-osteoblast MC3T3-E1 cells. In bone-forming cells, MAPK pathways are critical, as they target the RUNX2 protein to control osteoblast differentiation and osteogenic proteins, leading to bone formation [16,17,18,19]. The MAPK pathways and RUNX2 are also associated with autophagy, which occurs in osteoblast differentiation [20,21,22]. Soy B-mediated endoplasmic reticulum stress induces autophagy in colorectal cancer [23]. Autophagy is closely related to necroptosis, a type of programmed cell death [24]. Thus, we subsequently investigated its effects on osteogenic activity, osteogenicity, autophagy, and necroptosis to demonstrate the underlying biological mechanisms.

## 2. Results

### 2.1. Isolation and Identification of SoyB Purified from A. hypogaea

**Figure 1:**
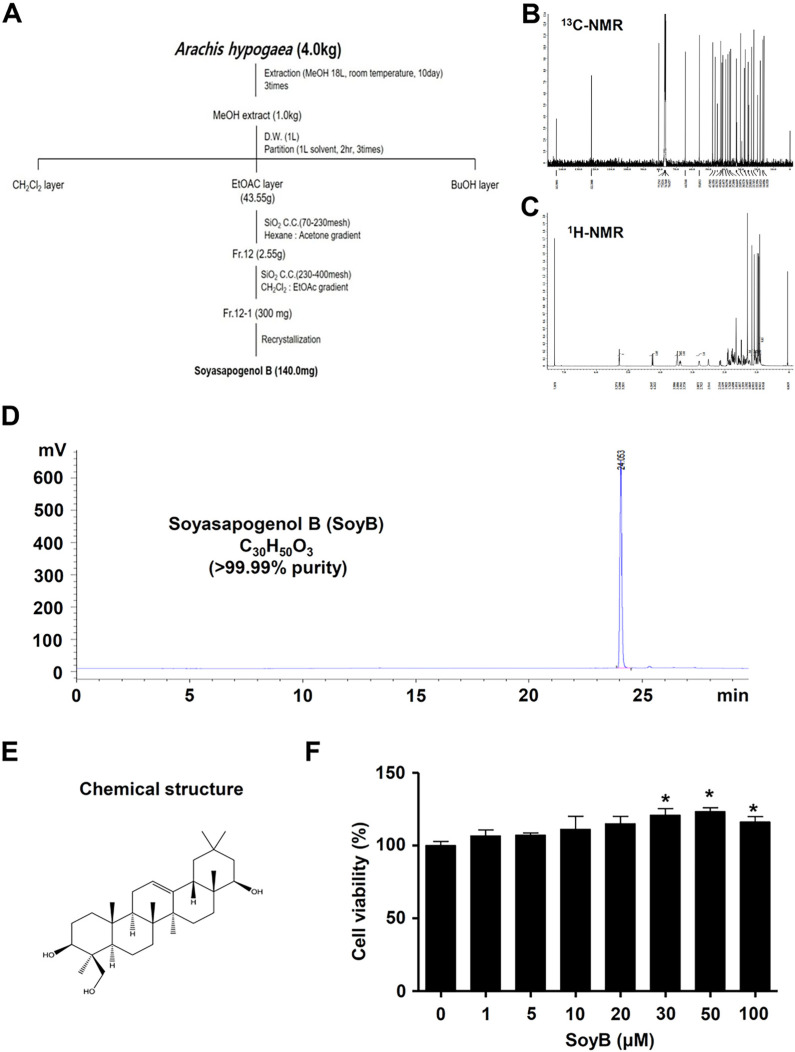
Isolation of SoyB from fruits of A. hypogaea and the effect of SoyB on cell proliferation (**A**–**C**). Isolation roadmap of SoyB from fruits of A. hypogaea (**A**), 13C NMR (**B**), and 1H NMR (**C**). Spectra (**D**,**E**): HPLC chromatogram, 99.9% purity (**D**), and chemical structure (**E**) of SoyB. (**F**) After MC3T3-E1 cells were seeded and treated with SoyB at concentrations of 1, 5, 10, 20, 30, 50, and 100 μM for 24 h, cell viability was analyzed using an MTT assay. Data are presented as the mean ± S.E.M. from three independent experiments. *, *p* < 0.05 was considered statistically significant compared to the control.

The fruit of *Arachis hypogaea* (4 kg) was extracted using MeOH (18 L, three times) at room temperature for 3 days. The crude extract (1000 g) was suspended in distilled water (DW) and then solvent-partitioned with CH2Cl2 and EtOAc. The EtOAc-soluble fraction was chromatographed on a silica gel column (Hexane:Acetone = 200:1) eluted with a stepwise gradient. Fr. 12 was rechromatographed on a silica gel column (CH2Cl2:EtOAc = 10:1) to obtain four fractions (Fr. 12-1~12-4). Fraction 12-1 was recrystallized from MeOH to create the compound (140 mg). The isolation roadmap is summarized in Figure 1A. The chemical structure was studied using an NMR analysis and was identified as SoyB by a comparison of the spectroscopic data with the previous literature [20]. EI-MS *m*/*z* = 458.72 [M]+. 13C-NMR (125 MHz, CDCl3) δ: 38.3 (C-1), 27.6 (C-2), 80.8 (C-3), 42.7 (C-4), 55.8 (C-5), 18.4 (C-6), 33.0 (C-7), 39.6 (C-8), 47.7 (C-9), 36.6 (C-10), 23.7 (C-11), 122.3 (C-12), 143.9 (C-13), 42.0 (C-14), 25.8 (C-15), 28.1 (C-16), 37.3 (C-17), 44.7 (C-18), 46.1 (C-19), 30.5 (C-20), 41.4 (C-21), 76.6 (C-22), 22.3 (C-23), 64.5 (C-24), 16.1 (C-25), 16.8 (C-26), 25.4 (C-27), 28.1 (C-28), 32.7 (C-29), 20.0 (C-30) (Figure 1B). 1H-NMR (500 MHz, CDCl3) δ: 5.28 (1H, m, J = 3.7 Hz, H-12), 4.24 (1H, d, J = 11.2 Hz, H-22α), 3.50 (1H, overlap, H-3α), 3.48 (1H, overlap, H-24a), 3.39 (1H, t, J = 11.0 Hz, H-24b), 2.79 (1H, d, J = 7.5 Hz, H-18β), 0.91~1.29 (each 3H, s, 7 × CH3) (Figure 1C). The HPLC chromatogram and the chemical structure of SoyB (white amorphous powder, molecular formula: C30H50O3; purity: >99.99%) are shown in Figure 1D and Figure 1E, respectively. We checked the UV MAX of the HPLC chromatography to confirm the purity of the SoyB and also checked the HPLC-ELSD as a result of checking with the chromatography (Supplementary Materials Files S1 and S2).

To investigate the cell proliferation effects of SoyB in MC3T3-E1 cells, the cells were treated with 1, 5, 10, 20, 30, 50, and 100 μM for 24, after which cell viability was analyzed using an MTT assay. It was found that 1–20 μM SoyB did not affect cell viability, but high concentrations (30–100 μM) significantly enhanced cell viability (1 μM: 106.7 ± 4.08; 5 μM: 107.2 ± 1.49; 10 μM: 111.2 ± 9.02; 20 μM: 115.1 ± 4.90; 30 μM: 120.9 ± 4.56; 50 μM: 123.3 ± 2.77; 100 μM: 116.2 ± 3.69) (Figure 1F). We further investigated the osteogenic effect of SoyB at a level of 1–10 μM with no cytotoxic effect on cell viability in MC3T3-E1 cells.

### 2.2. SoyB Enhances Osteogenic Activity and Maturation

**Figure 2:**
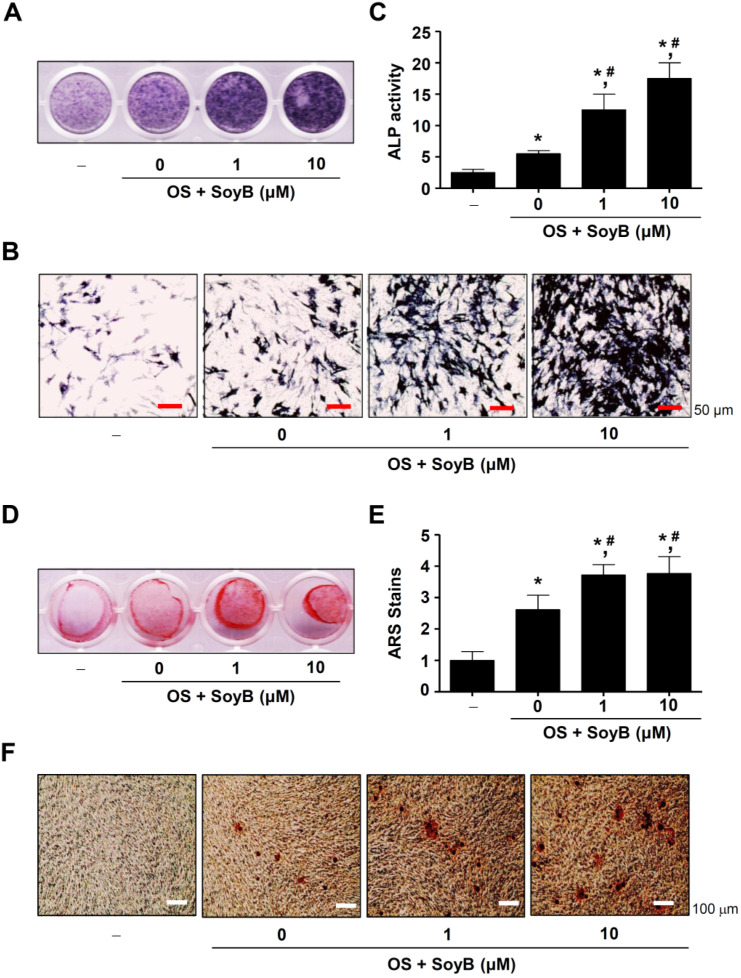
Effects of SoyB on early and late osteogenic activities. (**A**–**C**) After MC3T3-E1 cells were cultured in OS with SoyB at concentrations of 0, 1, or 10 μM for 7 days, early osteogenic activities were detected using ALP staining assays (**A**) and ALP enzyme activity (**C**) assays, and the individual ALP-stained cells were visualized under a light microscope. (**B**) Scale bar: 50 μm. (**D**–**F**) Late osteogenic activities were detected using ARS staining assay for 21 days (**D**), the ARS staining was quantitatively measured using a spectrophotometer (**E**), and the mineralization was visualized under a light microscope (**F**). Data are presented as the mean ± S.E.M. from three independent experiments. *, *p* < 0.05 was considered statistically significant when compared to the control. #, *p* < 0.05 was considered statistically significant when compared to OS.

To determine the osteogenic effect of SoyB, osteoblast differentiation was induced for 7 days in the absence and presence of SoyB, which was detected using ALP staining. It was found that SoyB increased the ALP stains (Figure 2A). The ALP positively stained cells were also observed using light microscopy (Figure 2B). Consistent with the results, it was found that SoyB significantly increased the ALP enzyme activity in a dose-dependent manner (Figure 2C). Next, we determined whether the presence of SoyB influencef the mineralization of the bone matrix from mature osteoblasts. Osteoblast differentiation was induced for 21 days to generate mature osteoblasts in the absence and presence of SoyB. An ARS staining assay was performed, and the results revealed that the presence of SoyB significantly improved the detection of the ARS stains (Figure 2D,E). Light microscopy observation also demonstrated that the presence of SoyB increased the mineralized nodule formation from mature osteoblasts (Figure 2F). These data suggest that SoyB promotes early and late osteoblast differentiation.

### 2.3. SoyB Enhances the Nuclear Expression of RUNX2 and Phosphorylation of Smad1/5/8 and JNK

**Figure 3:**
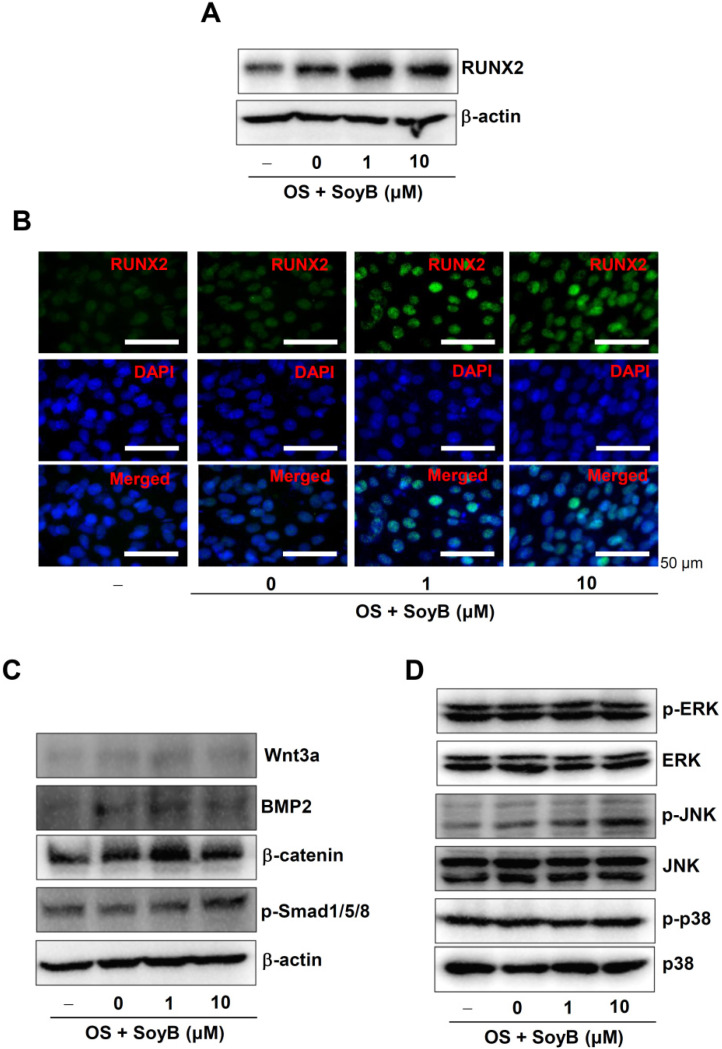
Osteogenic effects of SoyB on the RUNX2 expression and upstream molecules. (**A**,**B**) Total RUNX2 expression levels were assessed using Western blot analysis (**A**), and nuclear RUNX2 expression and localization were determined using RUNX2 (green) and DAPI (blue) staining and observed under a confocal microscope (**B**). Scale bar: 50 μm. (**C**,**D**) Equal amounts were probed using antibodies against Wnt3a, BMP2, β-catenin, phospho-Smad1/5/8 (p-Smad1/5/8), β-actin (**C**), phospho-ERK1/2 (p-ERK1/2), ERK1/2, phospho-JNK (p-JNK), JNK, phospho-p38 (p-p38), and p38 (**D**). Data represent the results of three independent experiments.

To determine the molecular mechanism underlying the osteogenic effects of SoyB, RUNX2 expression was investigated, since RUNX2 is a core transcriptional factor for osteoblast differentiation. Western blot analysis revealed that SoyB enhanced the expression of RUNX2 (Figure 3A). Next, the expression of RUNX2 in the nucleus after the treatment with SoyB was observed using an immunofluorescence assay. The results demonstrated that SoyB increased the levels of the RUNX2 positively stained cells in the nucleus (Figure 3B). SoyB treatment had no effects on bone morphogenetic protein-2 (BMP2), Wnt3a, and β-catenin, whereas SoyB increased the phosphorylation of Smad1/5/8 (Figure 3C). Next, we examined whether SoyB affects mitogen-activated protein kinase (MAPK) signaling. SoyB did not affect the phosphorylation of ERK1/2 and p38, whereas SoyB increased the phosphorylation of JNK. These data suggest that SoyB enhances osteoblast differentiation through RUNX2 expression and the activation of Smad1/5/8 and JNK.

### 2.4. SoyB Does Not Influences Autophagy and Necroptosis during Osteoblast Differentiation

**Figure 4:**
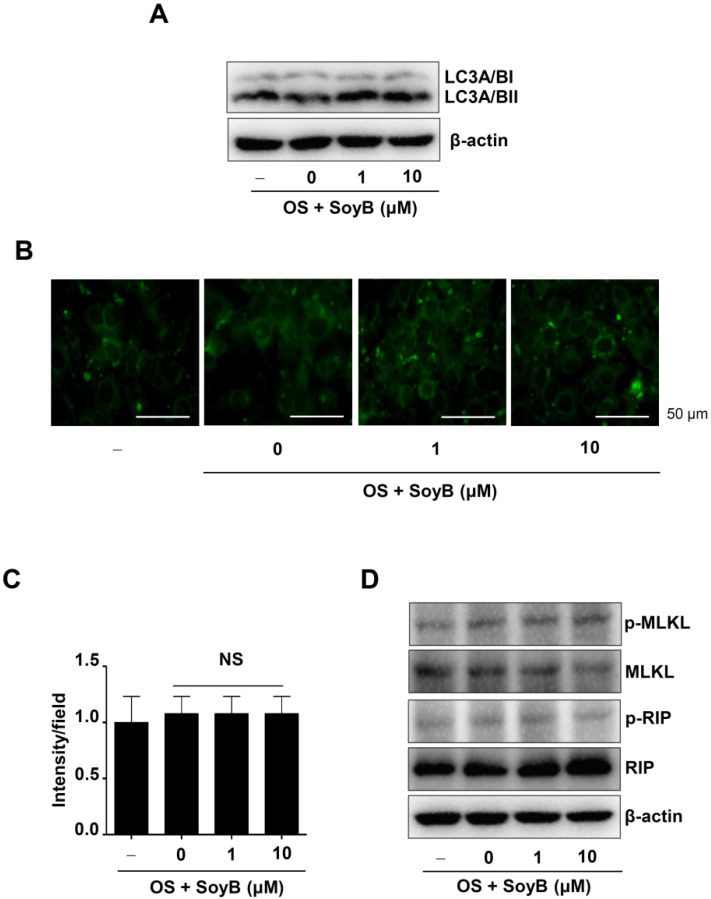
Osteogenic effects of SoyB on autophagic flux and necroptosis. (**A**–**C**) LC3A/BI and LC3A/BII levels were assessed by Western blot analysis (**A**), autophagosome formation was analyzed using DAPGreen Autophagy detection assay (**B**), and the intensity (fold) was shown as a bar graph (**C**). (**D**) Phospho-MLKL (p-MLKL), MLKL, phospho-RIP (p-RIP), RIP, and β-actin levels were assessed using Western blot analysis. Data are presented as the mean ± S.E.M. from three independent experiments. NS: not significant.

To determine whether SoyB affects autophagy during osteoblast differentiation, the expression and conversion of microtubule-associated protein light chain-3 (LC3) were detected, since LC3 is a widely used marker to monitor autophagy. Western blot analysis revealed that SoyB had no discernible effect on LC3A/B expression and LC3A/BII conversion (Figure 4A). DAPGreen was used to observe the autophagosome formation using an immunofluorescence assay. As shown in Figure 4B,C, SoyB also had no significant effect on the autophagosome formation. In addition, the molecular machinery of necroptosis was monitored, and the results revealed that SoyB did not discernibly affect the phosphorylation of receptor-interacting serine/threonine-protein kinase (RIP) and mixed-lineage kinase domains such as pseudokinase (MLKL) (Figure 4D). These data indicate that the osteogenic effects of SoyB are unrelated to autophagic flux and necroptosis.

### 2.5. SoyB Enhances Adhesion and Cell Transmigration during Osteoblast Differentiation

**Figure 5:**
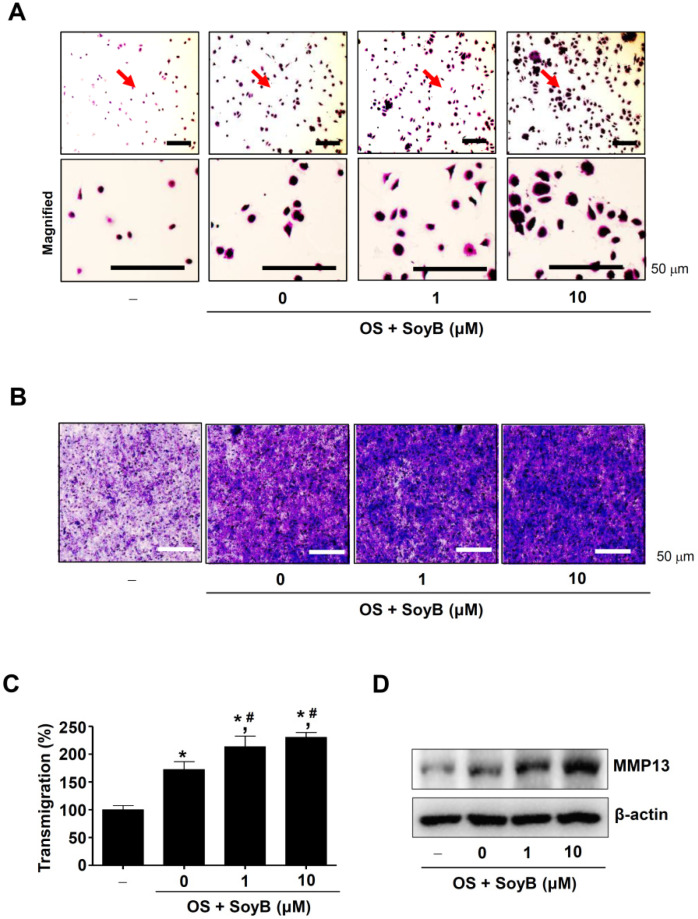
Osteogenic effects of SoyB on cell adhesion and transmigration. (**A**) SoyB-treated cells were seeded onto Matrigel-coated plates, fixed, and stained for 120 min. Cell adhesion was visualized under a light microscope. Magnified regions are indicated by red arrows. Scale bar: 50 μm. (**B**,**C**) Transmigration of SoyB-treated cells was observed using a light microscope (**B**), and the numeric values (%) are shown as a bar graph. Scale bar: 50 μm. (**D**) MMP-13 levels were assessed using Western blot analysis. *, *p* < 0.05 was considered statistically significant when compared to the control. #, *p* < 0.05 was considered statistically significant when compared to OS.

We subsequently investigated whether SoyB affects cell adhesion during osteoblast differentiation on Matrigel-coated 96-well culture plates. We found that SoyB increased the number of adherent cells with morphological stabilization when compared to the control and OS samples alone (Figure 5A). Next, a transmigration assay was performed using the Matrigel-coated polycarbonate filter in the Boyden chamber, and the results revealed that SoyB significantly increased the rate of cell penetration when compared to the control and OS samples alone (Figure 5B,C). In addition, SoyB increased the expression of matrix metalloproteinase-13 (MMP13), which plays an important role in the degradation of components in the extracellular matrix and is required for bone remodeling and repair (Figure 5D). Overall, these data suggest that SoyB is an enhancer for osteoblast differentiation, osteogenic activity, and maturation.

## 3. Discussion

*A. hypogaea* is widely used in foods and nutritional support. Most of the bioactive compounds (catechin, anthocyanidins, oleic acid, procyanidins, and epicatechin) from *A. hypogaea* have been associated with a reduced risk of cardiovascular diseases and cancers [25]. The present study is the first to demonstrate that SoyB purified from *A. hypogaea* enhances osteoblast differentiation by inducing osteogenic activity and bone matrix mineralization, without apparent autophagy and necroptosis. Osteoblast differentiation and maturation are required for bone formation and remodeling as well as for the bone repair process. ALP, which is a marker for early osteoblast differentiation, is a critical enzyme in osteogenic activity [26,27,28]. In the present study, we found that SoyB increased ALP enzyme activity and ALP staining levels to achieve early osteoblast differentiation. Moreover, SoyB promotes bone matrix mineralization during the late stage of osteoblast differentiation. As has been well established, inorganic pyrophosphates and organic phosphomonoesters are hydrolyzed by ALP enzyme activity, leading to the synthesis of hydroxyapatite which is provided for bone matrix mineralization [26,29]. It was reported that ALP-knockout mice showed spontaneous fractures, skeletal deformations, and areas of hypomineralization [29], thereby indicating that SoyB increases osteogenic activities to promote bone matrix mineralization from pre-osteoblasts.

RUNX2 is a core transcription factor that regulates gene expressions, including ALP, for osteoblast differentiation and bone matrix mineralization [30,31]. Based on the role of RUNX2 in ALP expression and osteoblast differentiation, we investigated RUNX2 expression and demonstrated that SoyB increases RUNX2 expression in the nucleus. It is well known that RUNX2 expression is controlled by BMP2/Smad1/5/8 and Wnt3a/β-catenin signals. In the present study, we demonstrated that SoyB has no effects on BMP2 and Wnt3a/β-catenin. However, we found that SoyB stimulates the phosphorylation of Smad1/5/8, or the downstream molecules of BMP2, and also the phosphorylation of JNK, but not ERK1/2 and p38. As has been well established, MAPKs (JNK, ERK1/2, and p38) play important roles in the regulation of RUNX2 protein, as well as in the major pathways that induce ALP expression, Smad1/5/8 and RUNX2 signaling [16,17,18,19]. Notably, JNK activation increases osteoblast differentiation and controls cell fate between the osteoblast and the adipocyte [32,33,34]. It has also been reported that JNK-mediated RUNX2 expression enhances osteoblast differentiation via a HOXA transcript antisense RNA, myeloid-specific 1 [35]. Thus, these findings indicate that SoyB induces RUNX2 expression through the activation of Smad1/5/8 and JNK to promote osteoblast differentiation and maturation.

Gradually, osteogenic studies have concluded that autophagy is involved in osteoblast differentiation. It was reported that rapamycin, an autophagic inducer, promotes osteoblast differentiation [20]. Deleting 200 kDa of focal adhesion kinase family-interacting proteins, which are an essential component of autophagy, has been shown to cause a significant decrease in bone mass and osteoblast differentiation [21]. A total of 10 μM Kaempferol, a flavonoid, induces autophagy to promote osteoblast differentiation and mineralization [22]. Additionally, 1 μM vitamin-K2-induced autophagy stimulates osteoblast differentiation and mineralization [36]. Previously, it was reported that SoyB regulates autophagy in colorectal cancer [23]. In the present study, we investigated the role of SoyB in autophagy and demonstrated that 1 and 10 μM of SoyB are not involved in the autophagic system during osteoblast differentiation. Next, we investigated a possible role of SoyB in necroptosis. Studies have revealed that tumor necrosis factor-a and ROS stimulate necroptosis in osteoblasts, and also that chronic ethanol consumption activates necroptotic signaling to stimulate osteoblast necroptosis, resulting in decreases in osteoblast differentiation and bone formation [37,38,39]. In the present study, we found that SoyB is not involved in the necroptotic system during osteoblast differentiation. Therefore, our data suggest that SoyB induces osteogenic effects through the activation of Smad1/5/8 and JNK and through the expression of RUNX2, regardless of autophagy and necroptosis.

Migration and adhesion are required for bone formation and bone repair. It was reported that MMP13 production in osteoblasts is involved in the degradation and remodeling of the extracellular matrix (ECM) during bone repair, and also is considered to play an important role in bone repair [40,41]. Toriseva et al. reported that the activity of MMP-13 may affect cell adhesion to the matrix and adjacent cells [42]. Cell adhesion is closely involved in the initiation and progression of cell proliferation and differentiation [43,44,45,46,47]. In the present study, we demonstrated that SoyB stimulates cell adhesion to ECM, as well as cell migration across ECM with the induction of MMP13. Thus, our findings provide convincing evidence that SoyB regulates osteogenic activities by enhancing osteoblast adhesion, migration, and subsequent differentiation.

In conclusion, to the best of our knowledge, this is the first study to report that SoyB promotes the adhesion and migration of pre-osteoblasts and subsequently stimulates osteoblast differentiation through the osteogenic Smad1/5/8 and JNK pathway and RUNX2 expression, regardless of autophagy and necroptosis. Our findings provide convincing evidence that SoyB has the potential to be useful for the development of a drug that modulates osteoblast differentiation and osteogenic activities in bone diseases.

## 4. Materials and Methods

### 4.1. General Experimental Procedures of Plant Material and Purity

Organic solvents used for extraction and partition, such as ethanol (EtOH), methanol (MeOH), n-hexane, ethyl acetate (EtOAc), and dichloromethane (CH_2_Cl_2_), were obtained from Duksan Chemical (Anseong, Gyeonggido, Korea). For the determination of their chemical structure, 1H-and 13C-NMR instrumental analyses were performed. The spectra were recorded on a Jeol ECA-500 spectrometer (Jeol, Akishima, Tokyo, Japan) at 500 and 125 MHz for 1H-and 13C-NMR, respectively. The chemical shifts were given in δ(parts per million) from the internal standard substance, tetramethylsilane (TMS). High-performance liquid chromatography (HPLC) spectrum analysis was recorded on an Agilent 1200 series (Agilent Technologis, Burnsville, MN, USA) using a photodiode array detector (PDA) and an evaporative light scattering detector (ELSD).

### 4.2. Cell Culture and Osteoblast Differentiation

Pre-osteoblast MC3T3E-1 cells (#CRL-2593, American-type culture collection, Manassas, VA, USA) were cultured at 37 °C in a humidified atmosphere of 5% CO_2_ and 95% air using α-minimum essential medium (α-MEM) (WELGEME, Inc., Gyeonggido, Korea) without L-ascorbic acid (L-AA) (Sigma-Aldrich, St. Louis, MO, USA) and supplemented with 10% fetal bovine serum and 1 X Gibco ^®^ Antibiotic-Antimycotic (Thermo Fisher Scientific, Waltham, MA, USA), as previously described [48]. The osteoblast differentiation of MC3T3E-1 cells was induced by changing to an osteogenic supplement medium (OS) containing 50 μg/mL L-ascorbic acid (L-AA) and 10 mM β-glycerophosphate (β-GP), as previously described [48]. The OS was replaced every 2 days during the incubation period. One hundred percent DMSO was used for the dissolution of SoyB, and the vehicle control was used at a final concentration of 0.1% DMSO.

### 4.3. Cell Proliferation Assay

3-[4,5-dimethylthiazol-2-yl]-2,5-diphenyltetrazolium bromide (MTT) assay was used to measure cell viability, as previously described [16]. Briefly, the cells were treated with MTT solution, incubated for 2 h, and then the formazan was solubilized using 100% DMSO. Absorbance was detected at 540 nm using the Multiskan GO Microplate Spectrophotometer (Thermo Fisher Scientific, Waltham, MA, USA).

### 4.4. Early and Late Osteogenic Activity Analyses

For the early osteogenic activity analysis, the osteoblast differentiation of MC3T3-E1 cells was induced for 7 days, and alkaline phosphatase (ALP) staining and activity tests were performed, as previously described [48]. Briefly, for the ALP staining assay, the cells were incubated for 1 h at 37 °C with an ALP reaction solution (Takara Bio Inc., Tokyo, Japan) and the level of ALP staining was observed using a scanner and a light microscope. To conduct the ALP activity assay as previously described [48], an alkaline phosphatase activity colorimetric assay kit (Biovision, Milpitas, CA, USA) was used and the ALP activity was quantitatively detected at 405 nm using the Multiskan GO Microplate Spectrophotometer (Thermo Fisher Scientific).

For the late osteogenic activity analysis, the differentiation was induced for 21 days, and an Alizarin red S (ARS) staining assay was performed, as previously described [48]. Briefly, cells were stained with 2% Alizarin red S (pH 4.2) (Sigma-Aldrich) for 10 min, then ARS staining was observed using a scanner and a light microscope, and then the staining level was quantitatively detected at 590 nm using the Multiskan GO Microplate Spectrophotometer (Thermo Fisher Scientific).

### 4.5. Western Blot Analysis

Osteogenic-, autophagic-, and necroptotic- regulatory protein levels and phosphorylation levels were analyzed using Western blot analysis, as previously described [49,50]. Briefly, protein concentration was detected using a Bradford reagent (Bio-Rad, Hercules, CA, USA). Equal lysates (20 μg) were analyzed using sodium dodecyl sulfate-polyacrylamide gel electrophoresis, polyvinylidene fluoride membrane (Millipore, Bedford, MA, USA), 1 × TBS containing 0.05% Tween 20 (TBST), and 5% skim milk. The specific primary antibodies were incubated overnight at 4 °C and horseradish peroxidase-conjugated secondary antibodies (1:10,000, Jackson ImmunoResearch, West Grove, PA, USA) were incubated for 2 h at room temperature. Protein signals were detected in the ProteinSimple detection system (ProteinSimple Inc., Santa Clara, CA, USA).

### 4.6. Immunofluorescence

An immunofluorescence assay was performed, as previously described [48]. Briefly, anti-RUNX2 antibodies (1:200, Cell Signaling Technology, Beverly, MA, USA) were incubated overnight at 4 °C and Alexa-Fluor 488-conjugated secondary antibodies (1:500, Invitrogen, Carlsbad, CA, USA) were incubated for 2 h at room temperature. Nuclei were stained using a DAPI solution (Sigma-Aldrich, St. Louis, MO, USA) for 10 min at room temperature. Eight-well chamber slides (Thermo Fisher Scientific) were mounted using a FluoromountTM Aqueous Mounting Medium (Sigma-Aldrich).

### 4.7. Autophagosome Formation Assay

The formation of autophagosome was detected using the DAPGreen Autophagy Detection Kit (Dojindo, Kumamoto, Japan) according to the manufacturer’s instructions. Briefly, cells were incubated with 0.1 μM DAPGreen solution, washed using culture medium, and treated with SoyB. The slides were mounted and autophagosomes were observed under a fluorescence microscope.

### 4.8. Transmigration Assay

A transmigration assay was performed using a Boyden chamber, as previously described [48]. Briefly, cells were incubated in the Boyden chamber with Matrigel (Corning Life Sciences, Tewksbury, MA, USA)-coated nuclear pore filters, fixed with 10% formalin, and stained with crystal violet. Transmigration was monitored using a light microscope.

### 4.9. Cell Adhesion Assay

A cell adhesion assay was performed, as previously described [50]. Briefly, cells were seeded onto Matrigel (Corning Life Sciences)-coated 96-well culture plates and adherent cells were fixed with 10% formalin and stained with crystal violet for 10 min. Cell adhesion was monitored using a light microscope.

### 4.10. Statistical Analysis

The statistical significance was analyzed using a one-way ANOVA with the Bonferroni test in the Prism Version 5 program (GraphPad Software, Inc., San Diego, CA, USA). *p* < 0.05 was considered statistically significant. All data are presented as the mean ± standard error of the mean (S.E.M.).

## Data Availability

The data that support the findings of this study are available from thecorresponding author upon reasonable request.

## Supplementary Materials

The supporting information can be downloaded at: https://www.mdpi.com/article/10.3390/ijms23158297/s1.

## Abbreviations

ALP	Alkaline phosphatase
ARS	Alizarin Red S
β-GP	β-glycerophosphate
ECM	Extracellular matrix
L-AA	L-ascorbic acid
LC3	Microtubule associated protein light chain 3
MAPKs	Mitogen-activated protein kinases
MMP	Matrix metalloproteinase
MSCs	Mesenchymal stem cells
MTT	3-[4,5-dimethylthiazol-2-yl]-2,5-diphenyltetrazolium bromide (MTT)
OS	Osteogenic supplement medium
RUNX2	Runt-related transcription factor 2
SoyB	Soyasapogenol B
