# Supplementary material for: Effects of Triterpene Soyasapogenol B from Arachis hypogaea (Peanut) on Differentiation, Mineralization, Autophagy, and Necroptosis in Pre-Osteoblasts
Source: Int J Mol Sci. 2022 Jul 27;23(15):8297. doi: 10.3390/ijms23158297 (PMC9368047; doi:10.3390/ijms23158297)
Supplement: Supplementary file 1 [file ijms-23-08297-s001.zip › Supporting Information file S2.pdf]

Data File D:\BOMI\DA...1PURITY\210923_`21PURITY(448-1009) 2021-09-23 18-07-54\210923_453.D
Sample Name: 210923_453

===

Acq. Operator : SYSTEM Seq. Line : 67
Acq. Instrument : 1260HPLC_ELSD2 Location : Vial 63
Injection Date : 2021-09-25 오전 4:16:16 Inj : 1
Inj Volume : 3.000 µl
Acq. Method : D:\BOMI\DATA\210923_`21PURITY\210923_`21PURITY(448-1009) 2021-09-23 18-07-54\PURITY_SOP.M
Last changed : 2021-09-24 오후 1:40:23 by SYSTEM
Analysis Method : D:\BOMI\DATA\210923_`21PURITY\210923_`21PURITY(448-1009) 2021-09-23 18-07-54\PURITY_SOP.M (Sequence Method)
Last changed : 2021-09-29 오후 2:12:18 by SYSTEM
(modified after loading)
Additional Info : Peak(s) manually integrated

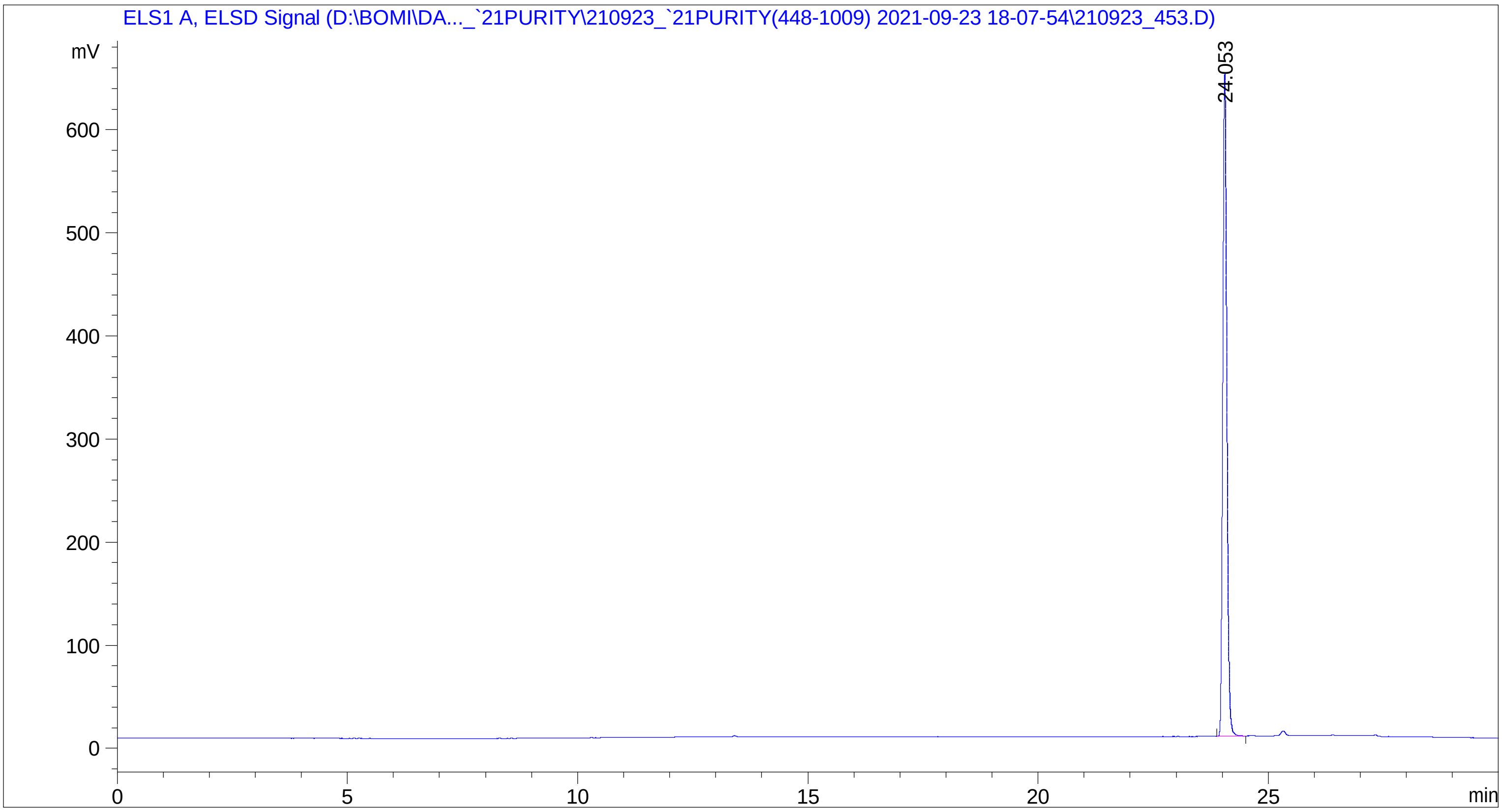

===

Area Percent Report

===

Sorted By : Signal
Multiplier : 1.0000
Dilution : 1.0000
Use Multiplier & Dilution Factor with ISTDs

Signal 1: ELS1 A, ELSD Signal

Peak #	RetTime [min]	Type	Width [min]	Area [mV*s]	Height [mV]	Area %
1	24.053	BB	0.0958	3955.41187	642.78937	100.0000

Totals : 3955.41187 642.78937

===

*** End of Report ***
