# Supplementary figures and images for: Effects of Triterpene Soyasapogenol B from Arachis hypogaea (Peanut) on Differentiation, Mineralization, Autophagy, and Necroptosis in Pre-Osteoblasts
Source: Int J Mol Sci. 2022 Jul 27;23(15):8297. doi: 10.3390/ijms23158297 (PMC9368047; doi:10.3390/ijms23158297)

□ \*DAD1, 23.895 (447 mAU, -) Ref=23.755 & 24.235

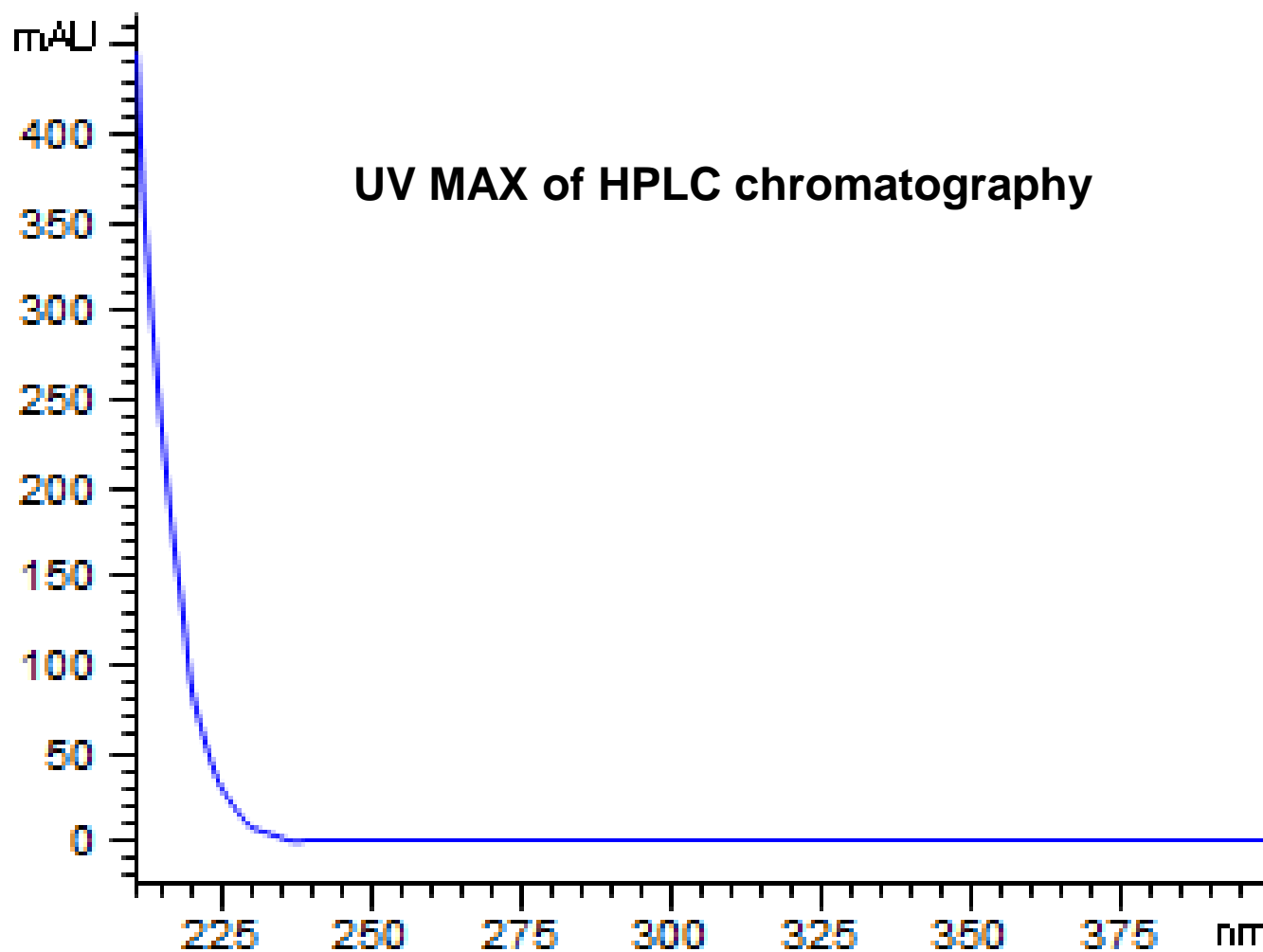

Supplement: Supplementary file 1 [file ijms-23-08297-s001.zip › Supporting Information file S1.pdf]
